# Effects of educational management on quality education in rural and urban primary schools in Ghana

**DOI:** 10.1016/j.heliyon.2023.e21325

**Published:** 2023-11-04

**Authors:** Ruth Donkoh, Wing On Lee, Ahotovi Thomas Ahoto, Josephine Donkor, Portia Oware Twerefoo, Martin Kudwo Akotey, Seth Yeboah Ntim

**Affiliations:** a Department of Education, Zhengzhou University, China; b Institute of Adult Learning, Singapore University of Social Sciences, Singapore; c University of Religions and Denominations, Qom, Iran; d OLEA M & G Insurance Brokers, Ghana; e Department of Public Administration and Management, University of South Africa, South Africa; f Department of Development Studies, Valley View University, Ghana; g Department of Psychology, Zhejiang Normal University, China

**Keywords:** Educational management, Quality education, Internet facilities, Rural schools, Urban schools, Education in Ghana

## Abstract

To ensure inclusive, equitable quality education, and encourage educational opportunities for lifelong learning worldwide; the United Nations set sustainable development goals (SDG) to achieve quality education. Thus, using SDG 4 quality education and system theory, this research seeks to identify the effects of educational management on quality education in rural and urban primary schools in Ghana. Moreover, the mediating role of internet facilities in educational management and quality education relationhsip has been discussed. Thus, 745 urban and 471 rural head teachers and teachers from Ghana participated in an online survey. The data was compiled and executed by structural equation model using SPSS-AMOS. The result reveals that educational management has a positive effect on quality education in urban and rural schools. In addition, urban schools have more quality education than rural schools. Although internet facilities have a positive effect on quality education their absence weakens the quality of education in urban and rural schools. Moreover, it is recommended that the Ministry of Education should arrange professional training for supervisors and head teachers to boost their monitoring and supervision strategies. Furthermore, Parents Teacher Associations can provide funding to support the monitoring and supervision activities to achieve success. Lastly, quality internet facilities should be built with limited charges in rural and urban schools.

## 1 Introduction

The global world has recognized educational management as the top priority in basic schools to achieve quality education [1]. UNESCO has persistently promoted educational management for over a decade and provided technical assistance to governments through capacity development, and methodologies to strengthen their educational management system [1]. Teachers and head teachers are leaders of educational management. They create the enabling school environment by ensuring and instilling discipline and identifying challenges, to ensure lifelong learning and academic success [2].

Research has discovered that countries in Sub-Saharan Africa, Arab states, and southern Asia experience poor educational management that has resulted in their record of 37.7%, 27.1%, and 20.6% respectively for illiteracy rate [3]. On the contrary, countries like Germany, the United Kingdom, and the United States of America have good educational management systems hence, their report shows that less than 7% of citizens are illiterates. Educational management in Africa has been attributed to the high enrollment rates in schools although at present the concern about educational management has shifted from operational management to quality of education [4]. The problem of poor quality education remains a major setback to education in many developing countries [5]. Quality education ensures the effectiveness and efficiency of the academic institution through the availability of material resources, the effectiveness of human resources, improvement measures, and compliance with the standard established [6]. Research has confirmed poor quality education in Ghana and other sub-Saharan African countries [7].

Many factors identified to impede quality education in Ghana and other African countries are educational management and technological gap [8]. In educational management, strategic planning, monitoring, supervision, and effective school discipline are critical issues [9], whereas the technological gap is the absence of internet connectivity, and technological equipment that hinders students search for global knowledge and reduces teachers scope to syllabi and textbooks, which are not favourable to the current learning needs [10]. Although researchers have explored the effects of educational management on quality education globally, these studies are usually conducted in developed countries such as America, Asia, and Europe [11]. However, few studies have explored the effects of educational management on quality education in Sub-Saharan African countries including Ghana. Moreover, the importance of internet facilities on quality education has also been overlooked [12]. Scholars have, therefore, called on the need to conduct empirical research on educational management and internet facilities with a focus on developing countries [13].

This research seeks to fill these gaps by focusing on Ghana, a developing country in sub-Saharan Africa [14]. Theoretically, this research has enriched and complemented the literature on the effects of educational management on quality education. Practically, this research has vital implications for educational managers and policymakers to improve the quality of education and achieve educational objectives in Ghana. This research, therefore, examines the effects of educational management on quality education using internet facilities as a mediating role in Ghana schools. The following research questions were addressed: (RQ1) What are the effects of educational management on quality education? (RQ2) What are the mediating effects of internet facilities on the relationships between educational management and quality education? The following sections discuss the literature review, the methodology, results, discussions, and conclusions.

## 2 Literature review

### 2.1 General perspective of educational management

Educational management is a key task and the pivot for the survival of any educational institution [15]. It involves a systematic process of strategic planning, monitoring and supervision, organizing resources, effective school discipline, drawing policies, and controlling activities to train pupils to be acceptable in the organization, institutions, and society [16]. Historically, different meaning has been given to educational management from several geographical locations in the world [15]. Educational management is also known as educational administration in some countries. Head teachers and teachers are responsible for educational management. The ability of the head teachers and teachers to effectively manage schools depends on their professional development and lifelong learning [2].

Educational management in Europe focuses on infrastructure, school improvement, finances, objectives and mission of the school, and the students well-being [17]. Conversely, educational management in Africa is based on a designed curriculum, laid down syllabus, and high enrolment. This type of educational management dominates globally, with scholars projection as the ultimate system of educational management [15]. This system of educational management is the bedrock that revolves around Asian institutions as it combines skills development with literature education [15].

Comparative studies between Africa and some developing countries on educational management have revealed many disparities in how the African educational system is managed [18]. Most African countries pay less attention to the need for educational management leading to students having a weak academic foundation and a high illiteracy rate in the continent [19]. Interestingly, some parts of Asia, America, and Europe have dedicated attention to educational management in primary schools. Though efforts are made to improve educational management in Africa, its efficacy is yet to be seen because performances in Africa cannot be compared to developed countries [20].

### 2.2 General perspective of quality education

Quality education is the diverse plans and reforms of the educational institution, intending to achieve efficiency and effectiveness by complying with the standards established [21]. It creates an opportunity to evaluate teachers pedagogies, students performances, and measures to achieve school effectiveness. It also entails the learning resources, technology, programme enrollment, completed modules, teaching methodology, professional qualifications of the teaching staff, co-curricular activities, performance awards, students' and teachers' perspectives on the institution’s operating management as well as their opinions and assessments of education [22].

At present the global world has given attention to quality education due to an increase in knowledge and technological advancement [2]. A person’s philosophy or “mindset” is influenced by what they learn, hence quality education is crucial. Consequently, it can be said that one’s education affects the way one lives and the decisions one makes every day. According to psychology, learning occurs when a subject’s conduct changes, indicating that they have become used to the material being taught.

### 2.3 General perspective of internet facilities in Ghana and other West African countries

Internet connectivity and technological equipment are crucial for users to achieve quality education in this present world. Internet facilities in education assess the availability of technological equipment for students and teachers to explore [23]. Internet facilities are a prominent factor in enhancing educational management.

Countries in Central Asia, Europe, and America have embraced its use by making data affordable, technological gadgets cheaper, and access to the internet a threshold capability [10]. However, this cannot be said about Ghana and other countries in Africa due to the cost of data [24]. Several schools in Ghana have little or no access to internet facilities; hence teachers and students have low internet facilities, which is creating a massive gap between schools in Africa, Asia, and Europe [25].

Studies have pointed out that many students from Africa are far behind in internet usage, impeding their ability to know what happens beyond their school and limiting them to their teachers alone [26]. The teacher’s poor internet efficacy harms their lesson notes' preparation [27]. Teachers' ability to research new teaching methods, and review practices in other parts of the globe is hampered by poor internet connectivity in developing countries [28].

Online education has become the new teaching model lacking in Ghana and some African countries due to poor internet facilities [29]. Educational platforms accessible through internet connectivity are unavailable to most schools in Ghana, as the traditional form of classroom learning is not being argued with new learning processes [25]. Currently, there are several online platforms where teachers can learn and attend conferences on teaching practices [19]. However, the issue of poor internet facilities and accessibility is depriving teachers in Ghana and other African countries of this resource [30].

## 3 Theory and hypothesis development

### 3.1 System theory

The system theory believes that relying on one aspect of what significantly affects a system, will not achieve results [31]. This concept is relevant in educational management as the provision of quality education can be assessed from the perspective of the system theory. The educational institution is an open system that manages itself by exchanging materials in the environment [32]. The environment provides the human resources, material resources, methodology, processes, and procedures of working together in an environment to achieve an output thus, quality education. Educational managers cannot operate in isolation but rather use the material and human resources to make the school curriculum a reality [32]. The head teacher and teachers are expected to manage the environment, the teaching staff, and students, and ensure the use of technological equipment. Using the system theory, the head teacher and teachers can communicate directives in the institution to facilitate the achievement of educational goals and objectives as scheduled.

### 3.2 Effects of educational management on quality education

Educational management remains vital to any educational system, directly affecting quality education [33]. Literature has classified educational management under three elements: monitoring and supervision, effective school discipline, and strategic planning. All these variables serve as pillars upon which educational management works [34]. Monitoring and supervision, demand that someone matches performance with established targets [35]. Monitoring and supervision positively affect quality education if appropriately done, similar to the effects of monitoring and supervision of other fields [33]. The relationship between monitoring and supervision of quality education has been positive in many studies as systematic monitoring helps to identify weaknesses and resolves them.

Literature has established strategic planning as the bedrock for an effective educational management system that yields quality education [36]. The quality of education experienced in Europe and America, which several nations emulated, was the result of solid strategic planning of the educational management system. It plans goals for each educational programme and sets a target that needs to be achieved [14].

Another aspect of educational management is effective school discipline, which is aimed at designing and putting up corrective measures to instill discipline in students [37]. The effects of school discipline on quality education remain subjects of research interest as some schools have prohibited several forms of discipline, and they perceived it as not having positive effects on quality education but rather making children timid [38]. From the above discussion, two hypotheses were developed.

H1a Educational management has positive effects on quality education in urban schools.

H1b Educational management has positive effects on quality education in rural schools.

### 3.3 Effects of internet facilities on quality education

Internet usage in educational institutions has grown tremendously. It is used to address a variety of educational objectives, including teaching, learning, and managing the educational process [39]. Internet facilities enhance quality education as they help meet the contemporary system of education by providing an opportunity for knowledge, information, educational resources, improving learning outside the classroom, and enhancing pedagogical processes by exposing teachers to the web for further research on concepts [39]. At present, internet facilities have transformed students processes of acquiring knowledge and more often than not, they rely on the network rather than what their teachers teach [39]. Students can decide what to learn, when to study, be able to learn in a supportive environment, seek help from colleagues and teachers, and share their acquired ideas and knowledge. While it is vital to keep in mind that the internet, of course, is not a panacea for all educational problems, it can help to unlock the human potential that can enhance teaching and learning. From the above discussion, two hypotheses were developed.

H2a Internet facilities have positive effects on quality education in urban schools.

H2b Internet facilities have positive effects on quality education in rural schools.

### 3.4 Mediating role of internet facilities between educational management and quality education

Internet facilities explain the availability of technological equipment to be used by teachers [40]. Currently, internet facilities have taken center stage in education as internet usage in education has gained more prominence than ever due to the growth in online education, which was influenced by the COVID-19 pandemic [38]. Studies are looking at access to the internet and the ability of the players in the educational sectors to use it to influence teaching and learning [38].

Internet facilities affect all areas of quality education due to their ability to connect different sectors, speed processing, expand learning coverage, and discover new things [38]. Internet facilities have positive effects on strategic planning, as it has become part of the long-term planning of several educational institutions to provide internet to teachers and students regularly [25]. Internet facilities also affect teamwork because they connect people at different geographical locations [25]. Internet facilities are a game changer in the educational sector; hence it has positive effects on most variables in the educational field [25]. From the above discussion, two hypotheses were developed.

H3a Internet facilities mediate the relationship between educational management and quality education in urban schools.

H3b Internet facilities mediate the relationship between educational management and quality education in rural schools.

### 3.5 Conceptual framework

**Fig. 1:**
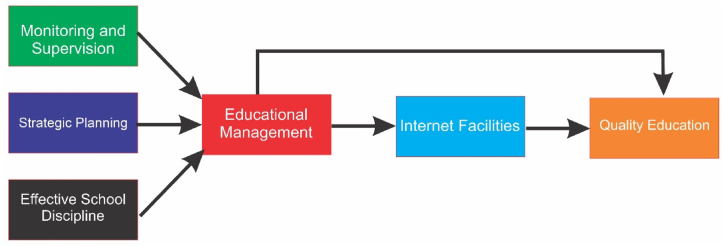
The conceptual framework of the study.

Based on the system theory and the hypothesis presented, a conceptual framework (refer to Fig. 1) has been designed to present the relationship between the variables.

## 4 Data and methodology

### 4.1 Sample and collection of data

This research is cross-sectional and employs a quantitative approach for data collection and analysis. The 745 urban and 471 rural teachers and head teachers participated from schools in Ghana. Using purposive sampling, data were extracted from teachers and head teachers in Ghana Education Service. As suggested by Ref. [41], a purposeful sampling technique was used based on the criteria: (a) context with regards to urban/rural setting due to profound differences in social and educational infrastructure in the two regions (b) availability of several basic schools, teachers, and head teachers to represent Ghana [41], and (c) few comparative studies conducted in Ghana give little attention to educational issues in the Upper East region due to enormous research funding gaps.

Moreover, using the stratified sampling approach enabled the researcher to select respondents representing the research population [42]. The researcher grouped teachers in classes one, two, and three into one stratum, teachers in classes four, five, and six into one stratum, and teachers from Junior High School form one to form three with their head teachers into different strata. This was because Ghana Education Service classified class one to class three as lower primary, class four to class six as upper primary, and Junior High School form one to form three as one unit. Based on the groupings, three strata were created in each region, leading to three strata representing rural area schools and three samples representing urban area schools.

Respondents for the study were selected based on the following criteria: they must be teachers or head teachers in primary schools in Greater Accra or the Upper West region. The teacher must be teaching in lower, upper, or junior high schools for not less than two years. The teacher must be certified by Ghana Education Services as a professionally qualified teacher willing to participate in the study. The questionnaires were distributed to the respondents using an online approach in March 2022. The online approach helped the researcher reach out to the respondents faster and at a cheaper cost [43]. The respondents were given 14 days to respond to the question. The 14 days were given to enable the respondents who were engaged with teaching and other duties to have the time to respond to the research questions at will. Telephone numbers and other contacts of the researcher and research assistants were provided. The respondents called to seek clarity on sections of the question and to seek explanations when the need arose. The participants who were not responding were constantly reminded through phone calls and emails to the district office and head teachers to motivate them about the value of the research and the need for them and their teachers to fill out the questionnaires [44]. The online data collection platform was protected with a password, which allowed only the researcher to have access to the data. After 14 days, a total of 98.2% of the participants responded to the questions on the online platform. In the end, 745 data were collected from the urban teachers, and 471 data were collected from the rural teachers.

### 4.2 Measures

The questionnaire had a 5-point Likert scale that ranged from 1 “strongly disagree” to 5 “strongly agree.” Studies have established the need for researchers to adopt or adapt instruments that have been satisfied in previous studies for data collection [33]. Based on that assumption, the researcher adapted instruments from similar studies for each construct used in the study.

#### 4.2.1 Educational management (EM)

The educational management constructs: monitoring and supervision (MS), effective school discipline (ESD), and strategic planning (SP) were measured by adapting questions from different studies. Based on the three constructs of educational management (MS, ESD, and SP); educational management was measured using a 17-item scale.

##### 4.2.1.1 Monitoring and supervision (MS)

The research adopted a 6-item scale (Cronbachs alpha = 0.876) that was developed by Ref. [45]. The items in this scale reflected monitoring and supervision in the organization that stimulates employees. MS constructs entail: *“The school has an effective supervision team who monitors activities periodically.”*

##### 4.2.1.2 Effective school discipline (ESD)

ESD scale was an adoption from Ref. [46] with six items, (Cronbachs alpha 0.913). ESD items involved “O*ur school has disciplinary committees”.*

##### 4.2.1.3 Strategic planning (SP)

Using [47], a 5-item (Cronbachs alpha 0.736) scale was used to measure SP based on organizational goals to achieve success. The SP constructs *entail “There are long-term plans that guide the school activities”.*

#### 4.2.2 Internet facilities (IF)

The research adopted [48] scale 5- items (Cronbachs alpha 0.813). The items reflected the influence of internet facilities on teachers and students. The IF constructs entailed “*there is no internet connectivity in our school”.*

#### 4.2.3 Quality education (QE)

QE used [49] with a 7-item (Cronbachs alpha 0.846). The scale measures factors that ensure quality education. The QE measures involved “*Our school has the right teachers”.*

### 4.3 Analytical procedures

To test the research- hypothesis, several analyses were conducted. Initially, we assessed missing data patterns with the title “missing completely at the random test” (*x*^2^ = 278.149, df = 269, p = .735) after which we treated the missing data using full information maximum likelihood recommended by Ref. [50]. Again, Harmans one-factor analysis was conducted to observe the presence of common method variance (CMV), and the results revealed 34.51% of the total variance, showing no common method bias problem.

Second, descriptive statistics, constructs correlation, exploratory factor analysis (EFA), and measurement model - utilizing confirmatory factor analysis (CFA) were performed to ensure the consistency of the data. Last, we tested our hypotheses with a hierarchical linear model (HLM) to estimate the direct effects of the variables. All analyses were conducted with IBM SPSS version 26 and IBM AMOS version 22.

### 4.4 Modelling

We estimate the models using probit regressions and their marginal effects since the dependent variable is binary. According to Ref. [51], the model uses the cumulative Gaussian normal distribution rather than the logistic function for calculating the probability of an event occurring or not. For robustness purposes, we used logistic regression for the estimations and found the results to be largely similar.

(1) $${QE=\beta }_{0}+Age\beta_{1}+Gen\beta_{2}+Educ\beta_{3}+Dur\beta_{4}+{Sch\beta }_{5}+Pos\beta_{6}+MS\beta_{7}+{ESD\beta }_{8}+{SP\beta }_{9}+\varepsilon_{i}$$

(2) $${IF=\beta }_{0}+Age\beta_{1}+Gen\beta_{2}+Educ\beta_{3}+Dur\beta_{4}+{Sch\beta }_{5}+Pos\beta_{6}+SP\beta_{7}+\varepsilon_{i}$$

(3) $${QE=\beta }_{0}+Age\beta_{1}+Gen\beta_{2}+Educ\beta_{3}+Dur\beta_{4}+{Sch\beta }_{5}+Pos\beta_{6}+ISE\beta_{7}+{EM\beta }_{8}+\varepsilon_{i}$$

QE-Quality Education.
IF- Internet Facilities.
$\varepsilon_{i}$- Error

## 5 Results

### 5.1 Demographic characteristics of respondents

Data were analysed using SPSS-AMOS version 22. Descriptive statistics presented tables showing background data with the variables' matching frequency, means, and standard deviations. From Table 1 below, the majority of 37.119% of the respondents were aged between 36 and 45 years, followed by 25.267% who were aged 26–35 years, signifying the youth age of Ghanaian teachers. Men dominated the teaching job in Ghana, as more than half of the teachers were male. The study shows that 65.6% of the respondents were men. In line with teachers' academic qualifications, most Ghanaian teachers hold a first degree, which is over 51% of them, followed by 42.2% who have diploma certificates in education. It must be acknowledged that the qualification of teachers in this study represents that of teachers in primary school, as teachers in higher educational institutions may hold higher qualifications. Most respondents (42.7%) have worked with the Ghana Educational Service for 6–10 years, with over 51% teaching in primary schools. Over 45% of respondents hold the position of subject teachers, while the rest are class teachers. The details are vividly stated in Table 1 below.

**Table 1:** Demographic characteristics of respondents.

Items	Groups	Frequency	Percentages	Accumulative
Age (Years)	18–25 years	112	9.218	9.218
	26–35 years	307	25.267	34.485
	36–45 years	451	37.119	71.604
	46–55 years	283	23.292	94.896
	Above 56 years	62	5.103	100
Gender	Female	418	34.403	34.403
	Male	797	65.596	100
Academic Qualification	Diploma	513	42.222	42.222
	Degree	623	51.275	93.497
	Postgraduate	79	6.502	100
Work Experience	Less than 5 years	314	25.844	25.844
	6–10years	520	42.798	68.642
	11–15 years	181	14.897	83.539
	16–20years	108	8.888	92.427
	Above 20years	92	7.572	100
School Level	Pre-school	213	17.530	17.530
	Primary school	623	51.275	68.805
	Junior high school	379	31.193	100
Job Position	Headmaster/Headmistress	110	9.053	9.053
	Departmental head	335	27.572	36.625
	Subject teachers	548	45.102	81.728
	Others	222	18.272	100

### 5.2 Analysis of data and results

Exploratory factor analysis was conducted on AMOS to check how the latent variables measured the construct. All loadings below the threshold of 0.6 were excluded from the analysis, while the factor loadings above the same threshold were included, as stated by Refs. [52,53]. The table below shows the loadings of latent variables concerning constructs. Educational management has three different constructs, namely: Monitoring and Supervision (MS), Effective School Discipline (ESD), and Strategic Planning (SP). Internet facilities and quality education have no sub-constructs. The results of the exploratory factor analysis are demonstrated in Table 2.

**Table 2:** Results of exploratory factor analysis for monitoring and supervision, effective school discipline, strategic planning, and internal consistency.

Latent Variables	Construct	Loadings	Cronbachs alpha	Composite reliability (CR)	Average Variance Extracted (AVE)
Monitoring and Supervision	MS_1	0.922	0.876	0.796	0.726
	MS_2	0.910			
	MS_3	0.783			
	MS_4	0.831			
	MS_5	0.859			
	MS_6	0.775			
Effective School Discipline	ESD_1	0.731	0.913	0.971	0.752
	ESD_2	0.824			
	ESD_3	0.783			
	ESD_4	0.713			
	ESD_5	0.831			
	ESD_6	0.751			
Strategic Planning	SP_1	0.947	0.736	0.956	0.738
	SP_2	0.893			
	SP_3	0.899			
	SP_4	0.928			
	SP_5	0.876			
Internet Facilities	IF_1	0.953	0.813	0.791	0.711
	IF_2	0.926			
	IF_3	0.923			
	IF_4	0.861			
	IF_6	0.728			
	IF_5	0.767			
Quality Education	QE_1	0.855	0.846	0.862	0.735
	QE_2	0.853			
	QE_3	0.881			
	QE_4	0.832			
	QE_5	0.764			
	QE_6	0.733			
	QE_7	0.731			

#### 5.2.1 Measuring internal consistency

The internal consistency of the constructs was measured using Cronbach alpha and composite reliability that produced results above the minimum threshold established by Ref. [54]. The Cronbach alpha of the constructs are as follows: effective monitoring and supervision recorded 0.796, effective school discipline 0.913, strategic planning 0.736, internet facilities 0.813, and quality education 0.846. On the other hand, composite reliability was used to solidify the internal consistency of the data and it produced results that signified a high level of internal consistency of measurement tools. The composite reliability ranges from 0.971 to 0.762 all are above the minimum threshold set by Ref. [55]. Average Variance Extracted (AVE) for the constructs ranges from 0.752 to 0.711 all above the minimum threshold of 0.5, indicating that the items used to measure the constructs explain more than 50% variance in the constructs [55]. The results are demonstrated in Table 2 below.

#### 5.2.2 Sample validity test

The sample adequacy, data dependency, and suitability were tested using Kaiser–Meyer–Olkin (KMO) and Bartlett’s test on SPSS. The test produced an acceptable result, above the minimum threshold of 0.5 with a significant level of 0.000. A Chi-square result of 1468.723 at 120 degrees of freedom, vital for good variable correlation was generated.

#### 5.2.3 Model fitness test

The model’s fitness to the factor pattern was conducted using the AMOS pattern matrix model builder, which the researcher used to confirm the study items. Variance techniques were used to govern the measurement describing the research items' latent components. The goodness of fit indices obtained is as follows CMIN/DF = 1.524 (1–3), TLI = 0.951 (>0.950), CFI = 0.963 (>0.950), and RMSEA = 0.047 (<0.05) with the Chi-square 268.67, degree of freedom of 104, and p-value = .00. The goodness of fit indices met the crucial levels. Table 3 shows that the goodness of fit indices met the crucial levels.

**Table 3:** Measurement models.

Fit Index	CMIN/DF	TLI	CFI	RMSEA
	1.524	.951	.963	.047

#### 5.2.4 Initial analysis: correlations between variables

Multiple correlations analysis was used to check relationships between variables, and hierarchical regression analysis was performed to test the hypothesis. The term “reliability” refers to a measurement’s consistency. Validity refers to the degree to which a measure’s scores accurately reflect the variable for which it was created [56]. All constructs employed in this study were tested for reliability using Cronbach’s alpha, factor loading, deduced from confirmatory factor analysis, and Average Variance Extracted **(**AVE). All variables used met the minimum threshold of all validity tests.

An initial analysis was conducted to check the correlation between the constructs of the study. From Table 4, the mean and standard deviation values of the constructs range from mean = 2.9980; SD = 0.53,887 for internet facilities, educational management has a mean of 3.0907; SD = 53,821, and quality education has a mean of 3.6046; SD = 0.81,506. Using correlation to show an initial relationship among composite variables, the result shows that most of the variables correlate with the highest correlation between internet facilities and educational management.

**Table 4:** Means, standard deviation, and inter-factor correlation analysis.

		Mean	STD	1	2	3	4	5	6	7	8	9	10	11
1	Age	3.35	.928	1										
2	Gender	1.39	.488	−.181**	1									
3	Edu.	2.95	.918	.225**	−.294**	1								
4	Duration	3.24	1.017	.784**	−.211**	.184**	1							
5	School	1.79	.407	.249**	−.300**	.335**	.275**	1						
6	Position	2.71	.924	.034	−.053	.191**	−.009	.138**	1					
7	QE	3.6046	.81,506	.128*	.088	.088	.143**	.187**	.066	1				
8	EM	3.0907	.53,821	.069	.037	.064	−.005	.088	.139*	−.066	.484**	.760**	1	
9	IF	2.9980	.53,887	.062	.023	.080	−.003	.083	.129*	−.084	.475**	.787**	.687**	1

*Key****:*** QE = Quality Education, EM = Educational Management, IF=Internet Facilities.

* = p < .05, ** = p < .005 and *** = p < .000.

#### 5.2.5 Testing the effects of educational management (monitoring and supervision, effective school discipline and strategic planning) on quality education in urban and rural schools using hierarchical linear regression analyses

From Table 5 below, the effects of all the three sub-constructs of educational management were used to predict quality education with the demographic variables used as control variables in modelD of Table 5. The Table contains two models. The “modelU” represents urban while modelR represents rural.

**Table 5:** Hypotheses testing the effects of educational management (monitoring and supervision, effective school discipline and strategic planning) on quality education in urban and rural areas schools using hierarchical linear regression analyses.

	ModelD	Model1U	Model2U	Model3U	Model1R	Model2R	Model3R
Variables	Quality Edu	Quality Edu	Quality Edu	Quality Edu	Quality Edu	Quality Edu	Quality Edu
	β(t)*	β(t)*	β(t)*	β(t)*	β(t)*	β(t)*	β(t)*
Constant	2.393***(7.922)	.956**(3.094)	.956**(3.094)	1.015**(3.290)	1.210***(3.917)	2.383***(7.394)	2.356***(7.308)
Age	.132(1.904)	.070(1.130)	.070(1.130)	.079(1.279)	.083(1.317)	.131(1.892)	.131*(1.896)
Gender	.033(.399)	−.008(-.104)	−.008(-.104)	.001(.008)	.016(.212)	.032(.381)	.029(.342)
Education	.010(.214)	.000(-.007)	.000(-.007)	−.007(-.176)	−.011(-.263)	.010(.209)	.009(.187)
Duration	−.127*(-2.039)	−.080(-1.452)	−.080(-1.452)	−.087(-1.570)	−.091(-1.612)	−.127(-2.038)	−.128*(-2.048)
School	.053(.512)	−.016(-.177)	−.016(-.177)	−.005(-.059)	.001(.015)	.052(.496)	.049(.464)
Position	.055(1.298)	.012(318)	.012(.318)	.017(.448)	.025(.656)	.055(1.295)	.055(1.299)
MS		.591***(9.239)			.520**(8.211)		
ESD			.557***(9.217)			.004(.088)	
SP				.017***(.448)			.016**(.325)
R^2^	.023	.023	.240	.233	.203	.024	.024
Adjusted R^2^	.004	.233	.224	.215	.185	.001	.001
R^2^	.065	.084	.6332	.7632	.5671	.322	.4510
F	1.203**	13.515**	13.516**	12.975***	10.892***	1.028	1.043

Key: *Model “D” = demography, Model “U” = Urban area school, Model “R” = Rural area school, β* = *beta, t = t-statistics in Planning.*

Hypotheses 1a and 1b examine the effects of educational management (MS, ESD, SP) on quality education in urban and rural schools in Ghana. The results of MS are presented in model1U and model1R in Table 5. The result shows that monitoring and supervision positively affect quality education in urban schools *(b = 0.591; p* < *.001*) and in rural schools (*b = 0.520; p* < *.01).* The results of ESD were presented in model2U and model2R, respectively in Table 5. The results show that effective school discipline has a statistically positive effect on quality education in urban Ghana *(b = 0.591; p* < *.001),* but the effects of school discipline on quality education in rural school is statistically insignificant. The results of SP were presented in model3U and model3R respectively in Table 5. The results show that strategic planning has positive effects on quality education in urban schools *(b = 0.017; p* < *.001)* and in rural schools *(b = 0.016; p* < *.01)*. Though strategic planning positively affects quality education in urban and rural Ghana, the statistical significance of its effect on education in urban areas is higher than in rural areas.

#### 5.2.6 Testing the effects of internet facilities on quality education

Hypotheses 2a and 2b examine the effects of internet facilities on quality education in urban and rural schools in Ghana. The results are discussed in model1U and model1R in Table 6. The result shows that internet facilities positively affect quality education in urban schools *(b = 0.082; p* < *.05)* but have no significant effects on rural schools. This indicated that hypothesis 2a is supported while hypothesis 2b is not.

**Table 6:** Hypotheses testing of effects of internet facilities on quality education using hierarchical linear regression model.

	ModelD	Model1U	Model1R
Variables	Internet facilities	Internet facilities	Internet facilities
	β(t)*	β(t)*	β(t)*
Constant	2.274***(9.127)	2.451***(9.297)	3.460***(15.296)
Age	.094(1.647)	.099*(1.738)	.103(2.212)
Gender	.033(.484)	.054(.784)	.035*(.616)
Education	.040(1.055)	.043(1.153)	.006(.183)
Duration	−.069(-1.342)	−.064(-1.259)	−.085*(-2.026)
School	.057(1.636)	.058*(1.671)	.048*(1.666)
Position	.100(1.163)	.128(1.472)	.072(1.021)
Quality E		.082*(1.976)	−.342***(-12.166)
R	.182	.213	.594
Adjted R^2^ rowhead	.014.	.023	.338
R^2^	.033	.046	.353
F	1.709	2.036*	23.328***

Key: *Model “D” = Demography, Model “U” = Urban area school, Model “R” = Rural area school, β* = beta, t = t-statistics in pretenses, * = *p* < .05, ** = *p* < .005 and *** = *p* < *.000, SP* = *Strategic Planning.*

#### 5.2.7 Mediating effects of internet facilities on the relationships between educational management, and quality education

In hypotheses 3a and 3b, the researcher examined the mediating effects of internet facilities on the relationship between educational management and quality education in urban and rural schools by bringing internet facilities into the relationship between educational management and quality education on model1M in Table 7. The result shows that internet facilities partially mediate the relationship between educational management and quality education. This is indicated by the change in a beta of educational management when internet facilities were added to the equation and the significant level of R^2^ change in the model1M of Table 7. Using a hierarchical linear regression model, the significant level of R^2^ change is a major indication of the mediation of a variable. The results are presented in Table 7 below.

**Table 7:** Hypotheses testing of mediating effects of internet facilities on the relationships between educational management and quality education using hierarchical linear regression model.

	Model 1	Model1M
Variables	Quality education	Quality education
	β(t)*	β(t)*
Constant	2.356***(7.308)	1.522***(4.511)
Age	.131(1.896)	.095(1.440)
Gender	.029(.342)	−.010(-.126)
Education	.009*(.187)	.015*(.342)
Duration	−.128(-2.048)	−.110(-1.858)
School	.049(.464)	−.014(-.137)
Position	.055(1.299)	.043(1.066)
IF		.678*(5.850)
EM	016*(.325)	.283**(4.132)
R^2^	.024	.124
Adjusted R^2^	.001	.101
R^2^ change	.024*	.101***
F	1.043*	5.291***

Key: *β* = beta, t = t-statistics in pretenses, * = *p* < .05, ** = *p* < .005 and *** = *p* < *.000, IF* = *Internet Facilities, EM = Educational Management.*

## 6 Discussions

The above results indicated that monitoring and supervision had influenced quality education in urban and rural schools in Ghana. Though there are little variations between the result of the monitoring and supervisions effects on education in urban and rural schools in Ghana, the outcome of this study is in line with several studies conducted on the subject in different countries [57].

Literature in West Africa presents mixed reports about the monitoring and supervision of education in rural areas in West Africa, with some stating less attention was given to schools in rural areas when compared to urban areas but much has not been established that rural schools were not monitored from time to time [37]. This study provides evidence that monitoring and supervision are effective tools to drive quality education in rural and urban schools in Ghana, as stated in other studies [58].

Comparatively, there is not much difference concerning the monitoring and supervision of education in urban and rural schools in Ghana, as Ghanas Education Service has enough circuit supervisors and officers stationed in many parts of the country to monitor schools' performance across the country [58]. This fact has also been reflected in our results and the findings by Ref. [58]. Findings of studies on education have indicated that monitoring and supervision remain key drivers of quality education in rural and urban areas [58].

The result also established that indiscipline remains a major challenge to quality education in rural areas. However, the study focused on the Ghanaian education sector, and the issue of disparities between school discipline in rural and urban areas in Africa remains a challenge to educational managers and other stakeholders [12]. The discipline of school pupils in urban areas remains effective due to parents' passion for education. In many cases, parents themselves deemed it fit for teachers to put all corrective measures in place to enforce student adherence to school rules in various urban schools [13]. Comparatively, many parents in rural areas in many African countries prefer to use their children for commercial activities, school farming, and trading on market days hence find it difficult to allow teachers to correct their children as several pupils are ready to stop the school should teachers enforce disciplinary measures [19].

The disparities between the effects of effective school discipline on quality education in rural and urban Ghana, as shown in this study, can also be articulated to poor interest in education in rural areas due to rural-urban migration [40]. The effects of increasing rural-urban migration in Ghana and other African countries are counting for rural schools not to be competitive. This has enabled stakeholders not to pay attention to some factors that drive quality education [19]. Most parents who want quality education for their children are quick to send them to schools in urban areas instead of helping to implement and enforce discipline in rural schools [19].

Though studies from advanced countries, America, and Europe indicated less effective relationships between school discipline and quality education, most studies in Africa prove positive effects between quality education and effective school discipline [37]. In a study in Africa, the poor relationship between quality education and effective school discipline in rural Ghana may be a major reason for poor student performance in these rural areas [37]. Nonetheless, the traditionally established evidence about school discipline has shown that it has influenced the quality of education, which is demonstrated in the high passing rate of students in effective discipline schools [37].

The results from the second hypothesis indicated that internet facilities have positive effects on education in urban schools. Most studies have established that internet facilities positively affect quality education in developed and developing countries [37]. The study result implies that internet facilities drive quality education which is equivalent to most studies around the globe. This gives more importance to internet facilities in education than other aspects of educational management [15]. The value of internet facilities concerning quality education is essential to several nations to the extent that internet facilities are made available, broadband is cheap, and affordable [36]. Further explanations of the effects of internet facilities on quality education also show that internet facilities in quality education encompass many activities that help draw an education road map for nations [36]. In a study conducted by Ref. [10], the researchers drew the link between quality education and internet facilities by stating that internet facilities involve the provision of technological equipment (projectors, television, computers, broadband, WIFI) to drive educational visions and missions [36]. Relating the above experts’ views to the findings of this study, it is clear that the failure of internet facilities can lead to the collapse of the entire educational system [28]. Hence, the finding of internet facilities influencing education in urban Ghana is an indication that when internet facilities are provided in rural areas the efficacy of the Ghanaian education system will be strengthened [8].

The finding of internet facilities not having a statistically significant effect on rural schools goes a long way to support an existing finding that has established the weak internet penetrations in schools in Africa [10]. The problem of weak internet facilities among educational workers and teachers emanates from poor access to internet services, expensive internet bundles, and the cost of technological gadgets [10]. Comparatively, schools in urban Ghana have access to internet services, which significantly affects the quality of education in these schools [36].

The poor internet facilities in rural schools affect teachers and other educational workers' ability to use the internet. Its limitations affect lesson note preparation in schools and are likely to affect online education, which is a major source of knowledge in schools [10].

Globally, the demand for internet usage is growing in schools, with several schools in both developing and developed countries using the internet to augment their teaching and learning tools [10]. A practical example of the effects of internet facilities’ usefulness is online education, which became the main measure of teaching and learning during the COVID-19 pandemic [23]. During the pandemic, schools in Asia and other developed countries engaged in online education to mitigate the effects of COVID-19 on their education system. Unfortunately, most countries in Africa had to close down [23]. The pandemic has created room for African countries to take a critical look at the need for online education; hence they have to put measures in place to enable educational workers, teachers, and students to have internet facilities for usage [23]. As stated in hypotheses 2a and 2b, there is a need to improve internet connectivity access in rural and urban schools to strategically enable teachers and students to benefit from internet usage [27].

The mediating effects of internet facilities on educational management and quality education is a further testimony of the role of internet facilities in today’s quality educational issues, as studies are clear that there cannot be quality education without access to internet connectivity, and teachers and pupils having improved skills on how to use the internet [23]. This proved the validity of the system theory that educational institution is an open system that manages itself by exchanging materials in the environment to achieve quality education [31].

To sum up the effects of educational management on quality education in urban and rural areas of Ghana comparatively, the results of the study have demonstrated that educational management influences quality education in all areas as all the three sub-constructs of quality education have positive effects on education in both urban and rural Ghana [7]. Except for effective school discipline, which has positive effects on quality education in urban areas, its effects on education in rural areas though positive, were statistically insignificant [12]. Internet facilities have the weakest effects on quality education as they weakly influence quality education only in an urban area school.

### 6.1 Theoretical implications

This present research contributes theoretically to existing literature. It provides proof that when monitoring and supervision are enhanced in schools, the academic performance of the pupil is likely to improve. Thus, improving monitoring and supervision helps to achieve quality education. Additionally, this research provides insight on how effective school discipline has promoted quality education in urban schools. More so, this research confirms that strategic planning has enhanced quality education in urban than rural schools which requires more strategic planning to be done in rural schools. Additionally, this research has confirmed that urban schools have weak internet facilities but the situation is worse in rural schools although internet facilities enhance educational management and quality education.

### 6.2 Practical implications for policymakers

This research has practical implications for policymakers. First, policymakers can use the research findings to improve the quality of education and achieve educational objectives in Ghana presently. Educational stakeholders can design timetables to ensure that monitoring and supervision teams and head teachers effectively monitor and supervise teachers’ work. The feedback from this activity needs to be discussed among the teachers and educational stakeholders to address the challenges in the school. Also, the Parents Teacher Association can provide funding and other materials as motivation to support the monitoring and supervision activities. Concerning managerial implications, the monitoring and supervision team and head teachers can be provided with professional training to acquire additional knowledge and skills that will polish their mode of monitoring and supervision to achieve the desired success. More so, a committee must be formed in the rural schools to effectively implement disciplinary measures. Finally, experts should be involved in the development of strategic plans for education, and strategic plans should be shared among all stakeholders for their suggestions before being implemented.

### 6.3 Limitations and future work

The limitations are that though the study is national in scope, the sample size was not large enough. The researchers mitigated these by developing outstanding criteria to enable them to select respondents that genuinely represent the population. The respondents were selected from different parts of the country so that no specific part was left out of the study. Since the study was a comparative study, the researcher had to present different results for different locations in the same table of analyses. This brought the problem of clarity of results. The researcher mitigated this by using the letter “R” to represent results for rural areas and the letter “U” to represent results for urban areas. This enables the readers to associate the right results with the correct settings.

Though the work was detailed enough, there is more room for future studies. The researcher suggested that future studies should use qualitative techniques to seek information from key players about improving education in rural areas. The research findings should be communicated to stakeholders in education in the future. Also, future work should concentrate on longitudinal data and panel data for better understanding. Finally, to enhance quality education in Ghana, the government should demonstrate its support through clear policy directives. Future studies can examine the initiatives and policy directives that the government through its Ministry of Education is implementing to ensure quality education in especially rural communities in Ghana.

### 6.4 Conclusion

The problem of poor-quality education in developing countries needs more research that propounds solutions to how the situation can be improved. Though some gains have been made over the past years, there are still gaps between the quality of education in rural and urban schools, a situation that has substantial adverse effects on school attendants and influences school dropouts.

The research investigated disparities in quality education in urban and rural Ghana by focusing on two significant variables of education. The researcher explores the constructs of educational management and internet facilities on quality education. All the results proved that the quality of education in urban areas is far better than in rural areas.

Some interesting revelations were made; internet facilities, a construct that details how internet connectivity is accessible, were explored to establish its effects on quality education. This research discovered that internet facilities promote quality education in urban areas but do not significantly affect quality education in rural areas.

Nonetheless, the study supports the narrative of poor-quality education in rural areas, and serious measures are needed to deal with the situation. Studies have revealed that previous steps have not yielded much-needed results and children’s difficulties in rural areas remain critical. The study has paved the way for further studies on the differences in quality education in rural and urban areas. Studies focusing on rural area education are needed in critical times like this, looking at the poor performance of children in rural schools.

### 6.5 Recommendations of the study

After analyzing the study results considering the current situation in Ghana and what has been established in previous studies, the following recommendation is made. Lack of internet connectivity in rural areas is not enabling effective teaching and learning; hence, the government, through the Ministry of Education, must look for ways to build a solid internet structure that will influence consistent internet connectivity at schools in rural areas at affordable prices. Nongovernmental organizations and other stakeholders should learn to provide internet-enabling innovative tools for teachers and students and teach them how to use them. Teachers and other stakeholders should be involved in the long-term strategic planning of educational issues. The educational plan at the governmental level should be broken down to the understanding of different stakeholders. Since rural education is lagging in terms of quality, the researcher proposed a separate unit of education planning focusing on rural areas. School discipline seemed to affect quality education positively but does not have similar effects on quality education in rural areas, indicating that discipline is ineffective in rural areas. Therefore, parents and teachers' associations must work out structures to discipline students who go wrong. There cannot be any effective education without discipline; hence, an effective discipline system must be instituted in rural area schools.

## CRediT authorship contribution statement

**Ruth Donkoh:** Conceptualization, Data curation, Formal analysis, Funding acquisition, Investigation, Methodology, Project administration, Resources, Writing – original draft. **Wing On Lee:** Conceptualization, Methodology, Supervision, Validation, Writing – review & editing. **Ahotovi Thomas Ahoto:** Conceptualization, Formal analysis, Methodology. **Josephine Donkor:** Conceptualization, Investigation, Methodology, Validation. **Portia Oware Twerefoo:** Formal analysis, Methodology, Validation, Writing – review & editing. **Martin Kudwo Akotey:** Conceptualization, Data curation, Investigation, Methodology, Validation. **Seth Yeboah Ntim:** Data curation, Formal analysis, Methodology, Validation.

## Declaration of competing interest

We, the authors of this manuscript declare no conflict of interest.
