# Supplementary material for: Effects of educational management on quality education in rural and urban primary schools in Ghana
Source: Heliyon. 2023 Nov 4;9(11):e21325. doi: 10.1016/j.heliyon.2023.e21325 (PMC10663754; doi:10.1016/j.heliyon.2023.e21325)
Supplement: Multimedia component 1 [file mmc1.docx]

**Appendix**

**Questionnaires for Data Collection**

**Zhengzhou University**

**Faculty of Education**

**Teachers/Headteachers Questionnaire**

These questions are to help study the effects of education management on quality education using internet self-efficacy as a mediator in urban and rural primary schools in Ghana. The study is an anonymous one and serves only academic purposes. Any information given would be treated as strictly confidential. Honesty in your response is very essential. You are invited to participate in this survey by completing the enclosed questionnaire. Thanks a lot.

**SECTION “A”: Demographic Information**

(1) Sex: (a) Male [ ] (b) Female [ ]

(2) Age (a) 25 years or below [ ] (b) 26-35 years [ ] (c) 36-50 years [ ] (d) 51 and above [ ] (3) What is your highest educational qualification? (a) Cert ‘A’ [ ] (b) Diploma [ ] (c) Degree [ ] (d) Masters [ ] (e) Doctoral [ ] Others, specify………………………………………………

(4) Number of years of teaching experience. (a) 1-5 years [ ] (b) 6-10 years[ ]

(c) 11-15 [ ] (d) 16-20 [ ] (e) 21 and above [ ]

**SECTION “B”**: **Education Management**

**Tick** where appropriate.

**Strongly Disagree (SD); Disagree (D); Neutral (N); Agree (A); Strongly Agree (SA)**

| **Educational management** | **ITEMS** | **SD** | **D** | **N** | **A** | **SA** |
| --- | --- | --- | --- | --- | --- | --- |
| **Monitoring and supervision** | 1. My head teacher does intensive monitoring and supervision. |  |  |  |  |  |
|  | 1. My head teacher’s management style enables monitoring of school activities in line with school’s objectives. |  |  |  |  |  |
|  | 1. The school has effective supervision team monitor activities periodically. |  |  |  |  |  |
|  | 1. There are key targets for which performance is monitored against. |  |  |  |  |  |
|  | 1. Authorities organize monitoring and supervision training for teachers and other stakeholders. |  |  |  |  |  |
|  | 1. Teacher and parent association members are involved in monitoring and supervision. |  |  |  |  |  |
|  | 1. There is motivation package for monitoring |  |  |  |  |  |
|  | 1. There is presentation of monthly or periodical report on monitory and supervision. |  |  |  |  |  |
| **Strategic Planning** | 1. There is long-term plans that guide the school activities. |  |  |  |  |  |
|  | 1. Teachers and other stakeholders are involved in designing the strategic plan. |  |  |  |  |  |
|  | 1. Teachers and other stakeholders have knowledge about the strategic plan. |  |  |  |  |  |
|  | 1. The strategic plans are reviewed periodically |  |  |  |  |  |
|  | 1. The strategic plan expands over three year and more. |  |  |  |  |  |
|  | 1. Actuals performance are compared to plan periodically. |  |  |  |  |  |
| **School Discipline** | 1. Our school has disciplinary committees |  |  |  |  |  |
|  | 1. There are stated code of conducts in our school. |  |  |  |  |  |
|  | 1. Students, teachers and other stakeholders were part of the development of the code of conducts. |  |  |  |  |  |
|  | 1. There are separate code of conducts for teachers and pupils. |  |  |  |  |  |
|  | 1. The school authorities are fair when applying disciplinary measures. |  |  |  |  |  |
|  | 1. Teachers and parent association members are involved in taking disciplinary decisions. |  |  |  |  |  |

**Section “C”: Internet Self-Efficacy**

**Tick** where appropriate.

**Strongly Disagree (SD); Disagree (D); Neutral (N); Agree (A); Strongly Agree (SA)**

| **Internet Self-Efficacy** | 1. Internet connectivity is poor in our school. |  |  |  |  |  |
| --- | --- | --- | --- | --- | --- | --- |
|  | 1. There is no internet connectivity in our school. |  |  |  |  |  |
|  | 1. Few teachers have access to smart gadgets that enables internet usage. |  |  |  |  |  |
|  | 1. We were educated on how to use the internet for teaching and learning. |  |  |  |  |  |
|  | 1. We practice on line learning sometimes. |  |  |  |  |  |
|  | 1. Data and internet services are too expensive |  |  |  |  |  |
|  | 1. The entire community where the school is has no access to internet services. |  |  |  |  |  |

**Section D: QUALITY EDUCATION**

**Tick** where appropriate.

**Strongly Disagree (SD); Disagree (D); Neutral (N); Agree (A); Strongly Agree (SA)**

| **Quality education** | 1. Our school has the right teachers. |  |  |  |  |  |
| --- | --- | --- | --- | --- | --- | --- |
|  | 1. Our school adhere to the right syllabus. |  |  |  |  |  |
|  | 1. Our school uses the right textbooks. |  |  |  |  |  |
|  | 1. Our students perform better in external examinations. |  |  |  |  |  |
|  | 1. Our students are able to express themselves in both English and local languages. |  |  |  |  |  |
|  | 1. Our students possess reading and writing skills. |  |  |  |  |  |
|  | 1. Our students sometime won awards. |  |  |  |  |  |
